# Reviewed article: Farias BO, Araújo LF, Tavares NHC, Oliveira BBR, Vasconcelos MSL, Machado SP. Intersectionality of gender, race/skin color and dental loss in adults: cross-sectional study with data from the 2019 Brazilian National Health Survey. Epidemiol Serv Saude. 2025:34;e20240524

**DOI:** 10.1590/S2237-96222025v34e20240524.a

**Published:** 2025-08-04

**Authors:** Arthur Cruz

**Affiliations:** Secretaria de Estado de Saúde de Mato Grosso do Sul, Escola Técnica do SUS

**Table:** 

Rating	Excellent	Good	Average	Below average	Poor
Interest	✓				
Quality		✓			
Originality		✓			
Overall		✓			

**Table:** 

Questionnaire	Yes	No	Not applicable
Does the manuscript contain new and significant information to justify publication?	✓		
Does the Abstract (Summary) clearly and accurately describe the content of the article?	✓		
Is the problem significant and concisely stated?	✓		
Are the methods described comprehensively?	✓		
Are the interpretations and conclusions justified by the results?	✓		
Is adequate reference made to other work in the field?	✓		

## Manuscript Structure

Length of article is: Adequate

Number of tables is: Adequate

Number of figures is: Adequate

Please state any conflict(s) of interest that you have in relation to the review of this paper (state “none” if this is not applicable): None

## Comments

Item 1:

Verifique se as referências citadas são relevantes e apoiam as afirmações feitas no texto. Reveja as referências: 30, 36, 37 e 38. Cada uma tem um detalhe a ser repensado.

A referência/citação 30 é de um estudo feito em uma cidade do interior de Santa Catarina, com cerca de 7 mil habitantes. E esse estudo não cita quantas pessoas foram avaliadas, além disso é um estudo qualitativo. Assim sendo, não podemos extrapolar essa informação à nível Brasil. Sugiro readequar o texto na discussão.

- Os autores defendem em vários momentos que pessoas pretas e pardas perdem mais dentes em comparação a brancos. Mas a população brasileira de pretos e pardos é de 54,5% enquanto de brancos é de 43,5%. Será que esses dados foram pareados? Ou calcularam apenas os dados brutos?

- As referências 36, 37 e 38 apresentam informações nas suas respectivas conclusões que não foram consideradas no texto do artigo em análise.

A referência 37 por exemplo, identificou que [... Aqueles (mulheres negras) que relataram saúde bucal regular ou ruim eram mais propensos a relatar tabagismo atual, perda dentária recente, diagnósticos de diabetes ou hipertensão.]

Ou seja, a perda dentária não está diretamente relacionada à cor da pessoa, está relacionada aos hábitos de vida (fumo e doenças metabólicas).

A referência 38 fez uma revisão sistemática com metanálise. A qualidade e quantidade de artigos analisados foram baixíssimos. E conclui que: [Os resultados aqui encontrados motivam o desenvolvimento de mais estudos para elucidar a influência da raça/cor de pele/etnia na hipótese estudada].

- A referência 32, citada nas Linhas 29 a 33 da página 5, cita as regiões Norte e Nordeste do brasil como as regiões que as mulheres pretas tendem a ter baixa renda, menor escolaridade, maior desemprego. Esta informação não concorda com os seus resultados apresentados na Tabela 1.

Sugiro discutir melhor essas informações. Porque aconteceu isto? Com a renda, escolaridade e localização regional.

Item 2: Rever a referência 30.

